# Registering New Drugs for Low-Income Countries: The African Challenge

**DOI:** 10.1371/journal.pmed.1000411

**Published:** 2011-02-01

**Authors:** Mary Moran, Nathalie Strub-Wourgaft, Javier Guzman, Pascale Boulet, Lindsey Wu, Bernard Pecoul

**Affiliations:** 1 Policy Cures, Sydney, Australia; 2 Drugs for Neglected Diseases initiative (DNDi), Geneva, Switzerland

## Abstract

Mary Moran and colleagues discuss the best strategies for African regulators to be supported in their efforts to evaluate and approve drugs for their own populations.

**Summary Points**

- A recent shift in the drug product environment for Africa has seen a score of new products being developed specifically for diseases of the developing world, creating new challenges for regulators in Africa and elsewhere. However, it is not at all certain that African regulatory authorities currently have the capacity to meet these new demands.
- The growing demand to assess novel neglected disease (ND) products for African use has generated a range of responses from policymakers and product developers, but there is limited guidance for product developers in choosing between approaches, and little or no integration between approval mechanisms.
- We discuss the various mechanisms in which novel ND drugs are assessed and approved for developing country use, and put forth six recommendations to create an efficient integrated system of national, regional, and international approvals to achieve an optimal drug registration approach for Africa that can reliably evaluate safety, efficacy, and quality of drugs for *African* use.

## Introduction

What is the best strategy to approve novel drugs for disease such as sleeping sickness that predominantly affect patients in Africa? How can African regulators best be supported to evaluate these drugs for their own populations? For many years, African medicines regulatory authorities (MRAs) have relied on stringent regulators in developed countries to assess novel pharmaceutical products such as drugs and vaccines for use in African populations. However, a recent shift in the drug product environment for Africa has put this approach under strain. A score of new products are now being, or have been, developed specifically for diseases of the developing world (Table 1), creating new challenges for regulators in Africa and elsewhere.

**Table 1:** Sample of novel neglected disease products presented to regulators since 2005 [8],[11],[12],[20]–[23].

Novel Neglected Disease Products	Regulatory Stage
Artesunate-amodiaquine ASAQ (malaria)	Approved by 24 African countries
	WHO prequalified (October 2008)
Artesunate-mefloquine ASMQ (malaria)	Approved by Brazilian ANVISA (April 2008)
Coartem Dispersible (malaria)	Approved by 14 African countries
	Approved by Swissmedic (December 2008)
	WHO prequalified (February 2009)
Intramuscular paromomycin (visceral leishmaniasis)	Received FDA and EMA orphan drug designation (March 2005)
	Approved by Drugs Controller General of India (August 2006)
Eurartesim (malaria)	Submitted to EMA for approval (July 2009)
Moxifloxacin (TB)	Clinical development plan submitted to developing country and/or Western regulators
PA-824 (TB)	Clinical development plan submitted to developing country and/or Western regulators
Arterolane/PQP (malaria)	Clinical development plan submitted to developing country and/or Western regulators
Azithromycin-chloroquine AZCQ (malaria)	Clinical development plan submitted to developing country and/or Western regulators
Fexinidazole (sleeping sickness)	Clinical development plan submitted to developing country and/or Western regulators

Additional source: correspondence with Novartis.

However, it is not at all certain that African regulatory authorities currently have the capacity to meet these new demands. A study conducted by the World Health Organization (WHO) in 2010 concluded that 90% of MRAs in sub-Saharan Africa “were in a situation which did not allow them to adequately carry out regulatory functions,” and thus could not guarantee the safety and efficacy of medicines to be used in their country [1]–[3]. While undoubtedly improving, growth in African regulatory capacity is not keeping up with these new challenges.

**Figure 1:**
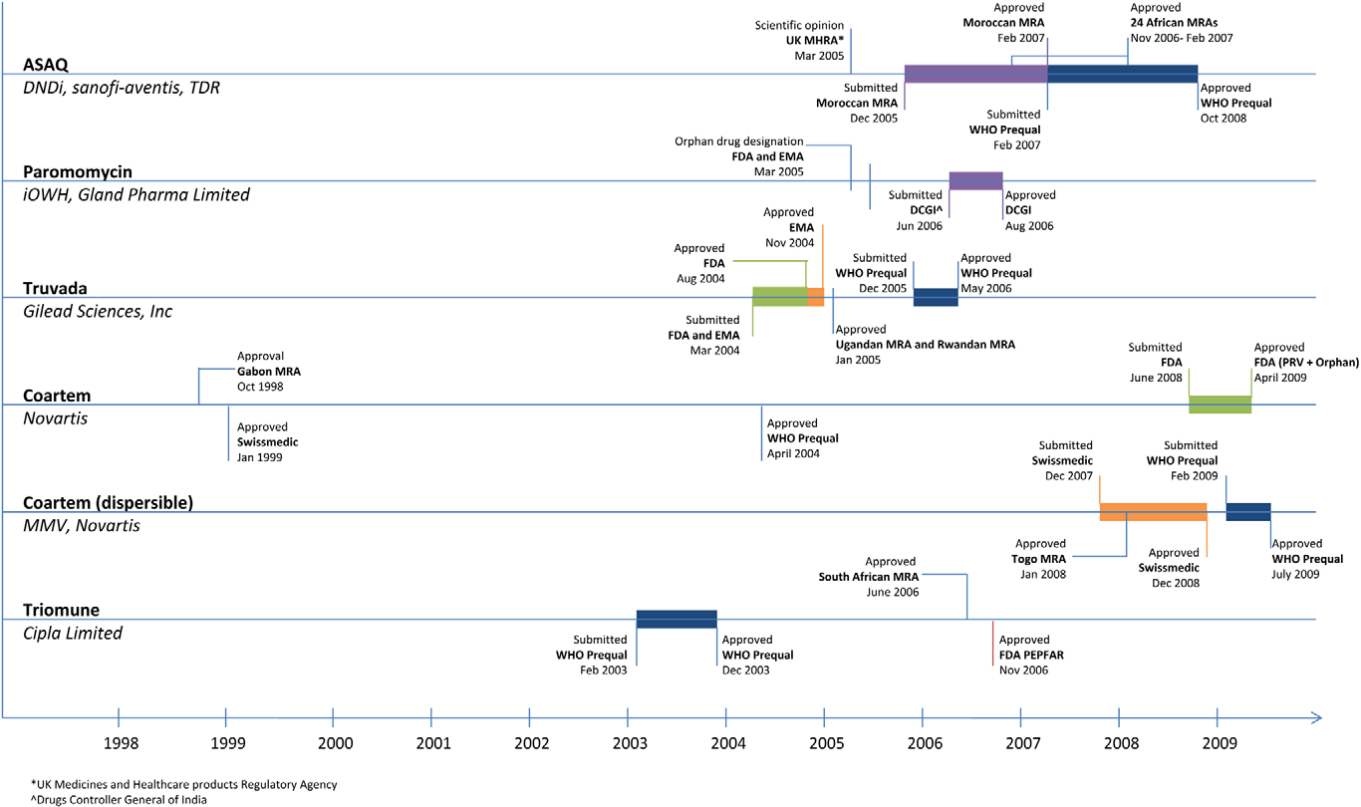
Neglected disease drug registration timeline **[7],[9],[11]–[19]**. Additional source: correspondence with Novartis.

The growing demand to assess novel neglected disease (ND) products for African use has generated a range of responses from policymakers and product developers, as outlined below. While each approach offers unique benefits, none is ideally suited as a primary vehicle for drug registration for Africa. There is also no guidance to product developers in choosing between approaches, and little or no integration between approval mechanisms (see Figure 1). It is now critical to review how novel ND drugs are assessed and approved for African use. This article is based on research conducted for a report titled “Registering New Drugs: The African Context” [4], commissioned by the Drugs for Neglected Diseases initiative (DNDi), and builds upon this work with additional research and analysis.

## Western Regulatory Approval Routes

Historically, the majority of new ND drugs have been first submitted to well-established Western regulatory authorities (e.g., United States Food and Drug Administration [FDA], European Medicines Agency [EMA], SwissMedic), either for routine regulatory review or under specific pathways such as Orphan Drug legislation (ODL) or expedited approval mechanisms. Multinational pharmaceutical companies and some Product Development Partnerships (PDPs) have typically used this approach because it offers clear protocols and rules, liability management and, in the case of ODL, tax breaks, free scientific advice, and market exclusivities. Firms also welcome the access Western regulatory approval provides to early commercial returns on products with overlapping rich and poor markets.

While bringing decades of regulatory experience to the table, use of Western authorities to review ND drugs also has drawbacks. It delays access for African patients since African MRAs often wait for the Western MRA decision before commencing action, and it puts ND product decisions in the hands of regulators who have less experience in tropical disease products, presentations, and epidemiology, and who are not accountable for the needs and safety of target African patients.

For instance, Western regulations may omit data requirements vital for safe large-scale use in Africa (e.g., trials assessing the safe interaction of HIV and malaria drugs). Rifapentine, a novel tuberculosis (TB) drug registered under US Orphan Drug provisions, ultimately could not be used in African TB patients despite being approved by the FDA because the trial design excluded HIV-positive patients. While HIV is less commonly associated with TB in the US, it represents up to 70% of TB patients in some sub-Saharan Africa countries, making the efficacy data submitted to the FDA inadequate for use in African populations [5]. Furthermore, the relative risk-benefit of ND drugs can be dramatically different in Africa and the West, where analysis against the same criteria can lead to completely different conclusions. For example, the first rotavirus vaccine, RotaShield, developed by Wyeth-Ayerst and licensed by the FDA in August 1998, was withdrawn from the US market in October 1999 due to a one in 10,000 risk of intussusception in children. This precluded its subsequent introduction in the developing world. While this risk-benefit analysis may have been valid for the US, where rotavirus causes less than 60 deaths per year, the vaccine was likely to have a much more favorable risk-benefit ratio in Africa, where rotavirus is responsible for approximately 5% of deaths in children under the age of five (a mortality rate of 183/100,000). Many of these problems are heightened in the case of regulatory pathways such as Orphan Drug approval and FDA Accelerated Review, which allow clinical trials to be abridged or downsized in order to expedite registration of treatments for diseases that are rare and life-threatening in the Western context (such as malaria), but affect millions of patients in the developing world.

## Neglected Disease–Specific Regulatory Pathways

Policymakers have responded to these shortcomings by developing regulatory pathways tailored for ND products, including the EMA's Article 58, WHO drug prequalification, and FDA “tentative approval”.

### Article 58

Article 58, established by the European Commission (EU) in 2004, aims to facilitate and assist developing country registration of medicines by providing the same scientific assessment (“opinion”) on products used outside the EU as for the EU, but incorporates WHO in the review process. Article 58's strength lies in its combination of stringent review standards, efficiency (average review time is 2.5 months), and structured input from WHO disease experts from disease-endemic countries. However, it has fallen victim to underutilization (only four product applications have been submitted since 2004), largely due to a lack of incentives for product developers to use this route. In particular, Article 58 does not offer tax breaks or market exclusivities; does not result in European marketing approval; is not linked to Orphan Drug approval; and does not formally expedite approval through WHO drug prequalification, although this may be changing.

### FDA PEPFAR-Linked Approvals

Following the launch of the US President's Emergency Plan for AIDS Relief (PEPFAR), the FDA introduced expedited approval in 2004 for HIV drugs purchased with PEPFAR funds for use outside the US. Seventy-one of the 100 products fully or tentatively approved (products still under patent in the US are given “tentative approval” until the patent expires) in association with PEPFAR as of June 2009 were generic formulations of existing drugs; 22 were new combinations or regimens of existing drugs not previously authorised in the US; and seven were pediatric re-formulations. The approval process is integrated with WHO prequalification through the exchange of reviews and the automatic inclusion of FDA-reviewed drugs in the WHO prequalification list: as of February 2010, 41% (113 drugs) of WHO prequalification drugs were PEPFAR approvals [6],[7]. While helpful and efficient in assessing non-novel HIV drugs associated with PEPFAR, this program's usefulness is limited by its disease and product restrictions.

### WHO Drug Prequalification

In 2001, the WHO began the drug prequalification program as a “surrogate” regulatory approval mechanism on which international procurement groups such as the Global Fund to Fight AIDS, Tuberculosis and Malaria could rely while developing country capacity for drug regulation was being strengthened. Evaluations are conducted by mixed teams of developed and developing country experts, with around one-third of reviewers from Africa. WHO prequalification has been relied upon by African MRAs as a proxy for their own drug assessments and approvals.

**Figure 2:**
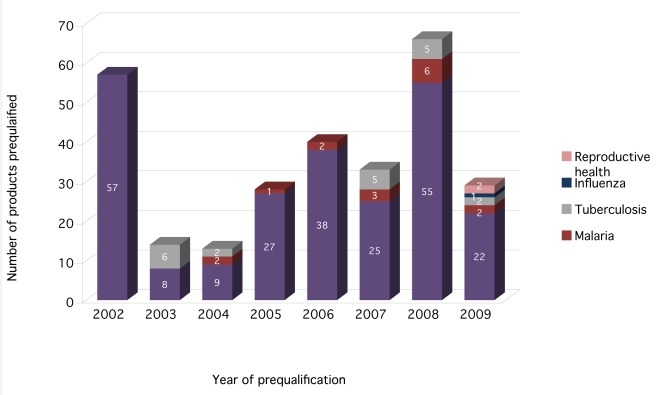
WHO prequalified drugs by disease [**7**].

WHO prequalification focuses on only a few diseases (in particular, HIV, malaria, and TB), with the majority of approved products being generic HIV drugs. As of June 2009, the program had pre-qualified 280 drugs—86% for HIV (241), 7% for TB (20), and 6% for malaria (16) (Figure 2). Just over half (56%) of these were generics, and 21% were new fixed-dose combinations or formulations of existing drugs. A further 23% were innovative drugs that had been approved by a stringent MRA prior to the WHO prequalification process.

WHO prequalification (in tandem with FDA tentative approval) has vastly accelerated African access to HIV, and to a lesser degree, malaria products; nevertheless, it could be further optimized. It covers only a few of the major diseases of Africa, and does not include a review of novel ND products. Due to its voluntary, no-fee, capacity-building approach, WHO prequalification can be slow (averaging 2 years) and it would benefit from more seamless integration with product reviews by stringent MRAs.

## Alternative Approval Strategies

In response to the drawbacks of both standard and ND-specific regulatory review, product developers have begun exploring alternatives, some of which offer insights for drug registration in Africa. Parallel approvals have been a common strategy for many PDPs, with dossiers submitted simultaneously to Western and developing country MRAs. The aim is to achieve high regulatory standards while expediting African registration. In practice, however, time gains are often illusory, as most African MRAs wait on WHO or Western approval before commencing their own process. Parallel approval also fails to assist or build the regulatory capacity of African MRAs.

Another potential strategy is twinned review, under which developing country regulators assess a pharmaceutical dossier in consultation with, or alongside, reviewers from stringent regulatory agencies. Twinned reviews can offer a potentially superior outcome by combining Western experience in product assessment with developing country expertise on endemic diseases, while expediting African regulatory approval and leaving risk-benefit analysis and decisions to MRAs responsible for areas where products will be used. More importantly, twinned review can build African MRA capacity through first-hand training for developing country regulators by Western regulatory experts. Nevertheless, there has not yet been a formal twinned regulatory review of any new ND product, although in 2008 the WHO organized a joint “practice” review of the artesunate-amodiaquine (ASAQ) dossier developed by the DNDi and involving regulators from African MRAs and the EMA. The implementation of twinned reviews will require resources and commitment by both Western and developing country regulators to move forward, but early stage joint reviews, such as those facilitated by the WHO with The Gambia, Mali, Ghana, and Senegal for the clinical trial application of the PATH Meningitis Vaccine Program's conjugate vaccine, are certainly a step in the right direction.

Product developers can also seek first approval from developing country MRAs without seeking prior, parallel, or twinned approval by WHO prequalification or a stringent regulatory agency. Used primarily by PDPs or developing country manufacturers, this option offers rapid access for domestic populations. For example, artesunate-mefloquine (ASMQ), developed by DNDi and Brazil's Farminguinhos/Fiocruz, was first registered in Brazil in April 2008 [8], and is currently under assessment by the WHO prequalification program. ASAQ, jointly developed by DNDi and Sanofi-Aventis, was first registered by the Moroccan regulatory authority in February 2007 and then received WHO prequalification in October 2008 [9], and the Institute for One World Health first registered intramuscular paromomycin for the treatment of visceral leishmaniasis in India in August 2006 [5].

## Discussion

An optimal drug registration approach for Africa should reliably evaluate safety, efficacy, and quality of drugs for *African* use. It should include African expertise, contribute to building African regulatory capacity, and, ultimately, expedite African access by reducing duplicative and sequential reviews by different regulators. However, as the above overview shows, the current system of ND drug approval is still far from achieving these goals. It is often inefficient, uses regulatory resources wastefully, and creates lengthy delays for patient access. Capacity-building opportunities for African regulators are routinely lost and, in the worst case, regulatory processes and decisions may not meet Africa's needs for the best, safest, and most appropriate drugs.

The following proposals are aimed at rapidly moving the current regulatory paradigm to the optimal scenario:

1. Institute formal twinned regulatory review; that is, any review of a novel ND product by a stringent MRA (or WHO prequalification) should formally include regulators from relevant endemic countries.
2. Automatic WHO prequalification of all novel ND products approved by stringent MRAs using standard regulatory pathways, and which meet WHO treatment recommendations. (With the exception of approvals under the Accelerated approval (FDA)/Conditional approval (EMA) mechanisms. Approvals under ODL should be reviewed on a case-by-case basis.)
3. Itegrate Article 58 with other approval mechanisms by allowing automatic WHO drug prequalification for products given a positive opinion under Article 58; AND allow positive Article 58 opinions to provide European market access either by conversion to EMA approval with a single European bridging study; OR link to automatic EU Orphan approval, which would additionally provide eligibility for tax breaks and market exclusivities.
4. Select experienced Western MRAs to conduct prequalifications on behalf of, and in addition to, the WHO.
5. Conduct a strategic review of WHO drug prequalification disease and product priorities, along the lines of WHO Strategic Advisory Group of Experts (SAGE) reviews for vaccines (established by the Director-General of the World Health Organization in 1999 to provide guidance on the work of the WHO Immunization, Vaccines and Biologicals Department), to identify additional priority diseases or products to be addressed by WHO prequalification (and/or outsourced to reference MRAs for prequalification).
6. Fund Centres of Regulatory Excellence in each of Africa's main regions that would conduct:
  - Joint review of product dossiers for the region (with external support as necessary).
  - Joint good manufacturing practices plant inspections for the region.
  - Clinical trial regulation, including joint regional review/approval.
  - “Twinned” reviews i.e., formal participation in external regulatory reviews such as FDA reviews, Article 58 assessments, or WHO prequalification.
  - Training and regulatory fellowships, including attachments to stringent external regulators and time with their national regulatory authority.

Collectively, these measures would improve the quality of ND drug reviews for the targeted populations; create an efficient integrated system of national, regional, and international approvals; expand the scope of regulatory support for Africa to include many more diseases and products; provide an institutional pathway to train and retain African regulators; and build African capacity to manage its own regulatory tasks. To move these ideas forward, it will be up to key policymakers in Africa and donor countries, funders of ND research and development, innovators, and, more importantly, regulatory agencies to reach a consensus on how these can be best implemented to ultimately benefit patients. The WHO, as a credible and trusted multilateral agency, can potentially play a large role in leading these efforts, as seen in recent pan-African initiatives such as the African Network for Drugs & Diagnostics Innovation [10].

In the face of scarce regulatory resources and large gaps in capacity, these proposals could address the immediate need for efficient, appropriate regulatory approval of new ND products, while building a sustained and independent African regulatory infrastructure in a way that truly addresses African needs and realities.

## Abbreviations

ASAQ: artesunate-amodiaquine
ASMQ: artesunate-mefloquine
AZCQ: azithromycin-chloroquine
DNDi: Drugs for Neglected Diseases initiative
EMA: European Medicines Agency
FDA: United States Food and Drug Administration
MRAs: medicines regulatory authorities
ND: neglected disease
ODL: Orphan Drug legislation
PDP: product development partnership
PEPFAR: President's Emergency Plan for AIDS Relief
TB: tuberculosis
WHO: World Health Organization
